# Case of Inflamed Hernia

**Published:** 1842-06

**Authors:** 


*Case of Inflamed Hernia.*—Prof. Williams exhibited to the Surgical Society of Ireland (Nov. 27, 1841) a very interesting pathological specimen, taken from a patient who had died a short time before in the city of Dublin Hospital, 76 hours after having been operated on for strangulated inguinal hernia. The individual in question, about 60 years of age, had labored under inguinal hernia of the right side for a period of six or seven years. He gave an unsatisfactory account of the antecedents of the case, but distinctly stated, that for the last four or five years he had worn a truss, which, however, retained the hernia very imperfectly, and that he frequently experienced much inconvenience from the hernia descending under, and being compressed by the pad of the truss. On admission to the hospital, he labored under mild symptoms of strangulated hernia, presenting very little indeed of urgency. Being a person of very limited intelligence, it could only be collected, in addition to what has been already stated, that he had been five or six days without having had a fæcal evacuation, during which time the hernia, previously reducible, could not be returned; he had kept his bed three or four days, and had vomited four or five times at long intervals. The hernial tumor was about half the size of the shut hand, it was by no means tense; and, when handled, the patient complained of uneasiness rather than of pain, and that to but a slight degree; there was very little abdominal tenderness on pressure, and the patient was able to lie on his back with the lower extremities stretched out at full length. The pulse was about 90, and rather small; tongue furred and moist; there was a good deal of thirst; and drink, which was only allowed to be taken in small quantities at a time, did not excite vomiting. The hernia could not be reduced, though, on attempting to do so, a considerable quantity of flatus was obviously expelled from the protruded bowel, and the tumor became more flaccid, and apparently slightly diminished in bulk, circumstances which, coupled with the (almost) absence of pain on pressure, caused the taxis to be persevered in longer than would otherwise have been done. Under these circumstances, small doses of sulphate of magnesia were administered by the mouth, and an enema directed to be thrown up every fourth hour. Two or three small fæcal evacuations occurred during the night, and, in the morning, matters were little altered, except that the patient stated, that he found himself, on the whole, more comfortable. Shortly after the visit, however, he began to vomit whenever he drank, and the tumor became painful, tense, and decidedly tender on pressure. Dr. O'Beirne's tube was now passed up the rectum, but as it failed to give relief, and the symptoms were rapidly growing worse, the operation for strangulated hernia was performed by Mr. Williams, in the evening.

On opening the hernial sac, which contained no fluid, and which was not adherent to the bowel at any part, the finger could be passed all around the neck of the sac, and through the inguinal canal fairly into the abdomen. The inguinal canal was very short, and its obliquity had quite disappeared, as often occurs in old hernia, according to a mechanism too well known to need description. There was, in fact, no stricture to free; no pressure or constriction was exerted by the rings, the inguinal canal, on the sac, or any portion of the intestine. Even now, however, the protruded bowel could not be returned until pressure was exerted on it for, perhaps, a couple of minutes; the obstacle to its return consisting in the condition of the intestine itself, its surface being covered with a thick layer of coagulable lymph, which rendered it rigid and unyielding; it was deemed essential, however, to return the bowel, as it gave the sensation to the touch, (which subsequently proved erroneous,) of containing some indurated mass, supposed to be fæces, in its cavity; shortly after the operation, the bowels were freely moved. The symptoms of peritoneal inflammation, however, continued despite the treatment, which chiefly consisted in the internal and external exhibition of mercury, and the patient died seventy-six hours after the performance of the operation.

On dissection, the coats of the portion of the intestine which had been protruded (the ileum about twelve inches from its termination) were found to be enormously thickened—in some parts fully to the extent of a quarter of an inch. The proper tissues of the bowel were not hypertrophied; the thickening depended on the effusion of coagulable lymph, partly situated on the serous surface of the intestine, partly in the subserous cellular tissue. In consequence of this condition of things, the calibre of the intestine was diminished to less than a fourth of its natural capacity; and it was the deceptive sensation thereby communicated to the touch which had led to the belief that indurated fæces were impacted in the bowels. The serous coat of the intestine was inflamed, and covered at points with gelatinous lymph; but, as manifestly appeared from the inspection of the preparation, the intensity of the inflammation diminished as the distance increased from the portion of intestine that had constituted the hernia. The inflammation had evidently commenced at that point, the morbid alterations being there at their maximum, and had thence extended, becoming less intense as it spread. The parietal layer of the peritoneum was not inflamed. A portion of the abdominal parietes and scrotum, comprising the entire of the sac and of the inguinal canal, had been removed, and were exhibited, whence it clearly appeared that no stricture or compression could have been exerted on the intestine by any of these structures.

Professor Williams said that it might be collected from what he had stated, that he considered the operation had been in this case, to say the least, useless. He nevertheless, gave the case publicity. This had clearly been a case of *inflamed* hernia, and not a case of *strangulated* hernia. But how was this difference to be diagnosed during life? He confessed he did not know. The diagnosis of this distinction, a most important one, had yet to be established. In this case, the point of departure of the inflammation was, beyond all dispute, the protruded portion of the intestine; the preparation placed that beyond doubt, and yet, till within a few hours of the operation, that portion of the intestine was, he might fairly say, almost indolent.—*Am. Jour. Med. Sci.*, from *Dublin Medical Press*, Dec. 29, 1841.

